# Monitoring Fatigue Damage of Modular Bridge Expansion Joints Using Piezoceramic Transducers

**DOI:** 10.3390/s18113973

**Published:** 2018-11-15

**Authors:** Tianyong Jiang, Yaowen Zhang, Lei Wang, Liang Zhang, Gangbing Song

**Affiliations:** 1 School of Civil Engineering, Changsha University of Science and Technology, Changsha 410114, China; tianyongjiang@csust.edu.cn (T.J.); zywcsust@163.com (Y.Z.); leiwang@csust.edu.cn (L.W.); zlcsust@163.com (L.Z.); 2 Department of Mechanical Engineering, University of Houston, Houston, TX 77204, USA

**Keywords:** Lead zirconate titanate (PZT), modular bridge expansion joints (MBEJs), fatigue damage, wavelet packet-based energy, active Sensing

## Abstract

Modular bridge expansion joints (MBEJs) are commonly used in bridges and are often subjected to fatigue damages, which necessitate fatigue monitoring of MBEJs to ensure the reliable operation of the bridges. In this paper, a stress wave based active sensing approach using piezoceramic transducers is developed to monitor the fatigue damage of MBEJ. A MBEJ involves mainly center beam, edge beam, support bar, support box, sliding bearing, sliding spring, elastomeric strip seal, full-penetration weld and reinforcing plate. In practice, for a MBEJ, the part that is most prone to fatigue damage is the full-penetration weld between the center beam and the support bar. In this paper, a specimen, which is the full-scale center-beam/support-bar (CB/SB) assembly, was designed and fabricated to facilitate the experimental study. The assembly mainly includes center beam, support bar, reinforcing plate, and full-penetration weld. The lead zirconate titanate (PZT) transducer bonded on the support bar was used as the actuator and the PZT transducer mounted on the center beam was as the sensor. Dial indicators were utilized to measure the vertical displacement of the center beam. Two series of tests, including static test, and fatigue test, were performed on the specimen in an alternating fashion. Based on the number of cyclic loading, the experiment was divided into six different stages: 0th cycle (the healthy state), 0.8 million cycles, 1.6 million cycles, 2.4 million cycles, 3.2 million cycles, and 4 million cycles. The signals received by the PZT sensor were analyzed with the help of wavelet packet analysis. In addition, the structure stiffness also was considered as a comparative approach in this paper. Experimental results show that during the fatigue test, the structure stiffness decreases with the number of cycle loading. However, the method can only obtain the fatigue damage impact on the entire structure, and cannot determine the fatigue damage degree of a certain weld. On the other hand, the proposed method can accurately monitor the fatigue damage degree of full-penetration welds. The research results show that the developed piezoceramic enabled active sensing approach can monitor and estimate the fatigue damage in MBEJ in real-time.

## 1. Introduction

The MBEJs have been used on the large-span bridges to accommodate high movements and support large dynamic wheel loads [1,2,3]. In addition, the MBEJs can protect the underlying superstructure from rainwater infiltration. The joints are subjected to more load cycles than other superstructure elements, since the wheels act directly on them. Therefore, the MBEJs are especially susceptible to fatigue damage as they undergo the cyclic loading from the axle of the vehicles [4,5]. The fatigue damage significantly reduces the service life of the MBEJs, and may even cause structural failure. To reduce and avoid economic losses caused by the fatigue damage, it is necessary to develop reliable techniques to monitor and evaluate the fatigue damage of the MBEJs.

Monitoring the fatigue damage of the civil engineering structures has been actively investigated [6,7,8]. The current methods for monitoring the fatigue damage mainly include Barkhausen noise measurements [9,10,11,12], X-ray tomography [13,14,15,16], metal magnetic memory (MMM) testing method [17,18,19,20,21], acoustic emission (AE) [22,23,24,25], ultrasonic and nonlinear ultrasonic techniques [26,27,28,29,30], among others. Barkhausen noise measurements were used to evaluate fatigue damage of engineering structural steel structures [9]. X-ray tomography is a technique that can be applied to visualize the internal structure of materials. Marrow et al. demonstrated the feasibility of imaging short fatigue cracks with high resolution synchrotron X-ray tomography [13,14]. The metal magnetic memory testing method is a novel nondestructive testing technology developed to monitor crack propagation and predict fatigue life [17,18]. The acoustic emission (AE) monitoring has been developed as an effective method for the detection, location and monitoring of fatigue damage in the metal structures by monitoring the acoustic signature of the fatigue crack during the opening/closing process [22,23]. The ultrasonic technique is a traditional method used for fatigue damage detection by using ultrasonic transducers [26] and, the nonlinear ultrasonic techniques, that have higher detection accuracy, are also commonly used [27,28,29,30]. Though strain based on methods have been used to monitor fatigue crack in welds [31], recent years have seen the development of strain based smart skin for fatigue crack detection [32,33]. In addition, reliability methods and statistical analyses are often used in bridge fatigue assessment based on monitoring data [34,35,36]. 

In structural health monitoring, PZT transducers, with the advantages of low cost, wide bandwidth, strong piezoelectric effect, and sensing and actuating abilities, have been extensively researched [37,38,39,40,41]. Many researchers have utilized PZT transducers to monitor the fatigue damage based on the wave propagation method [42,43,44,45,46,47] and electromechanical impedance (EMI) technique [48,49,50,51,52]. Ihn and Chang developed the wave propagation method using built-in PZT sensors/actuators to monitor fatigue crack growth in metallic structures [42]. Soh and Lim demonstrated the feasibility of fatigue damage detection and characterization using the EMI technique with surface-bonded PZT patch [48]. Li et al. performed the online monitoring of the fatigue crack initiation and propagation stage with the EMI method. The results demonstrated that the EMI method was very sensitive to minor damages and the entire process of crack initiation, crack propagation, and unstable fracture during fatigue loading [52].

**Figure 1:**
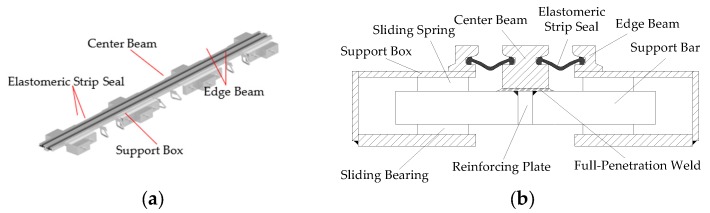
The MBEJ. (**a**) Three-dimensional view. (**b**) Main components.

In this work, we propose a stress wave based active sensing method using piezoceramic patch transducers to evaluate the fatigue damage in the modular bridge expansion joints with full-penetration weld in real-time. The MBEJ adopted in this paper is shown in Figure 1. The MBEJ involves mainly the center beam, the edge beam, the support bar, the support box, the sliding bearing, the sliding spring, the elastomeric strip seal, the full-penetration weld and the reinforcing plate. The center beam is rigidly connected to the support bar with the full-penetration weld. To strengthen their connections, the reinforcing plates are welded along the longitudinal direction of the support bar, therefore the support bar and the center beam can act as a whole sliding within the support box through the sliding bearing and sliding spring. Additionally, the edge beams are welded directly to the top of the support box, and they act as a whole while moving with the structure. In practice, in a MBEJ, the part that is most prone to fatigue damage is the full-penetration weld between center beam and support bar. Most of the fatigue damages originated in this connection zone [3,53,54].

To experimentally monitor the fatigue damage, a full-scale CB/SB assembly of the MBEJ as a test specimen was adopted in this paper. The PZT transducers with compression mode was bonded on both sides of each full-penetration weld of the test specimen. One of the PZT patches mounted on the support bar was used as an actuator to generate the stress wave, and the other one mounted on the center beam was utilized as a sensor to receive the responding signal. The dial indicators were installed to monitor the vertical displacement of the center beam. The hydraulic pulsation fatigue testing machine was utilized to load on the test specimen Compared with the fatigue damage monitoring research that involve only single component in the literature, the research involves the full-scale CB/SB assembly that consists many full-penetration welds, which differentiates this research with those related to fatigue damage monitoring in the literatures. In addition, this research applied the different static loading levels after finishing a certain cycle number on the test specimen, which enabled the paper to perform a comparative study on the influence of fatigue damage to the structure stiffness and to the signals received by the piezoelectric sensors. The experiment study demonstrates the advantage of the developed piezoceramic transducer enabled active sensing method to monitor the fatigue damage of the full-scale CB/SB assembly of the MBEJ in real-time.

## 2. Fatigue Damage Detection Principle

### 2.1. Active Sensing Approach

In this research, a PZT-transducer enabled active sensing approach is developed to monitor the fatigue damage of the modular bridge expansion joints. The fatigue damage often originates from the full-penetration weld between the center beam and the support bar. To present the active sensing approach, the following illustrates the fatigue damage example of the test specimen.

**Figure 2:**
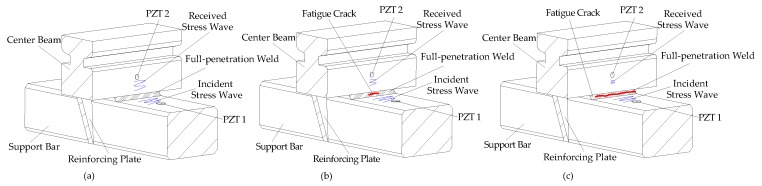
The principle of the active-sensing approach. (**a**) The initial state before fatigue loading. (**b**) Fatigue crack occurs in the weld throat. (**c**) Fatigue crack grows along the longitudinal axis of the center beam.

The principle of the active-sensing approach is illustrated in Figure 2. One PZT patch bonded on the support bar, as an actuator, is used to generate a stress wave, and the other PZT patch mounted on the center beam, as a sensor, is utilized to detect the wave response. Some defects are unavoidable in full-penetration weld, including undercut, overlap, pit, incompletely filled weld, and surface cracks, among other. The signals are received by a sensor at the initial state before fatigue loading, as shown in Figure 2a. When the MBEJ is under the fatigue load, these defects tend to generate stress concentration as the source of fatigue damage. With the number of fatigue cycles increasing, the fatigue damage in the throat of the weld expands, until it develops into a fatigue crack, and the signals received by a sensor decrease, as shown in Figure 2b. As the fatigue loading cycle further increases, the fatigue stress in the throat of the weld continues to accelerate the fatigue cracking in the narrow full-penetration, the received signal will continuously decrease until the fatigue loading process is complete, as shown in Figure 2c.

### 2.2. Wavelet Packet-Based Energy Analysis

Fatigue damages significantly affect the stress wave propagation in civil engineering structures [55]. The fatigue damage characterization of the MBEJ in the early age should be quantitatively evaluated. Applications of wavelet packet-based energy analysis in structural health monitoring have been investigated in recent years [56,57,58,59]. In the wavelet packet-based energy analysis, the sensor signal can be decomposed by n-level wavelet packet decomposition into 2*^n^* frequency bands. The signal energy of each frequency band can be computed by the summation of the square of each sampling data in the frequency band. Further, the total of the signal energy is obtained by the summation of the signal energies from all the frequency bands. In this research, the wavelet packet-based energy analysis helps to quantify the energy of the signal received by PZT transducers. The energy index of the received signal can be established for the purpose of directly comparing the change of the received signal energy during the fatigue loading process. In the wavelet packet-based analysis, the sensor signal X can be decomposed by a *n*-level wavelet packet decomposition into 2*^n^* frequency bands. *X_j_* can be expressed as:

*X_j_* = [*X_j,1_*, *X_j,2_*, …, *X_j,m_*] (1)

where *m* is the number of sampling data and *j* is the frequency band (*j* = 1, …, 2*^n^*). Additionally, the total energy of the decomposed signal *E_i,j_* can be defined as:

*E_i,j_* = ||*X_j_*||*^2^* = *X_j,1_^2^* + *X_j,2_^2^* + … + *X_j,m_^2^* (2)

where *i* represents the data measured in different time. The total signal energy at time *i* can be computed by the summation of the decomposed signal energy *E_i_*:

*E_i_* = [*E_i,1_*, *E_i,2_*, …, *E_i,2_^n^*] (3)

In this research, since fatigue damage often occurs at the weld joint, two PZT patches were mounted on both sides of weld joint to monitor fatigue damage in real-time. In the healthy state, the original signal energy (*E_0_*) was collected, and the signal energy collected was recorded as *E_i_* in different time. With the increase of fatigue times, the fatigue damage increases gradually at the weld joint, and the signal energy *E_i_* received by the piezoelectric sensor will continuously decrease. Therefore, this paper can analyze the fatigue damage state through utilizing the changes of the signal energy *E_i_* in the fatigue test.

## 3. Experimental Investigation

### 3.1. Specimen Details

**Figure 3:**
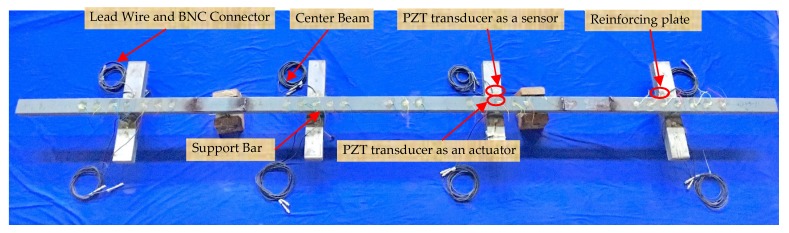
The photo of the full-scale CB/SB assembly of the MBEJ.

The test specimen was a full-scale CB/SB assembly that is a representative of that used in actual engineering applications, as shown in Figure 3. In the assembly, the full-penetration welds were utilized as the connections between the center beam and the support bars, and the reinforcing plates mounted along the longitudinal direction of the center beam were to strengthen these connections.

**Figure 4:**
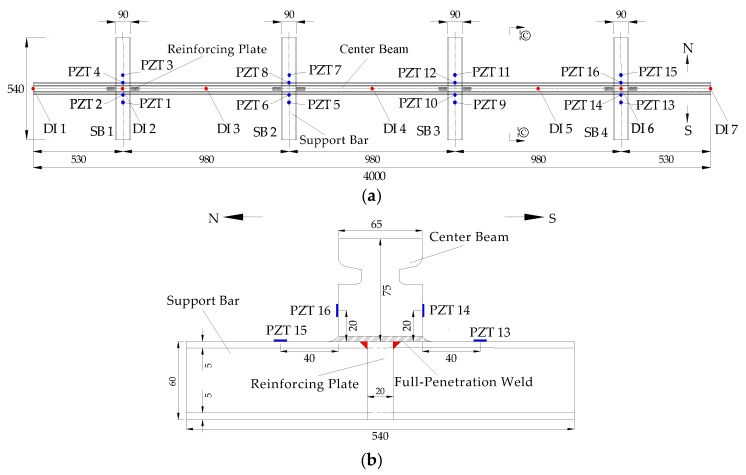
The details of the test specimen (unit: mm). (**a**) Top view. (**b**) I-I section left view.

The details of the test specimen are shown in Figure 4. The center beam with a length of 4000 mm in the test specimen was connected by the support bar with a length of 540 mm. The distance between the support bars was 980 mm. The center beam was a rectangular cross-section with the groove, which was applied to install the elastomeric strip seal. Its width and height were 65 mm and 75 mm, respectively. The support bar also was a rectangular cross-section with a width of 90 mm and a height of 60 mm. These parts adopted ASTM 572 Grade 50 (Q345B) steel, and its material properties were shown in Table 1. The Young’s modulus and shear modulus were 200 GPa and 76.9 GPa, respectively. Its yield stress and ultimate stress were 350 MPa and 450 MPa, respectively.

In this research, to study the fatigue damage of the full-scale CB/SB assembly specimen, two PZT patches with compression mode was mounted on both sides of each weld joint along the longitudinal axis of CB, as shown in Figure 4. One of the PZT patches mounted on the support bar was used as an actuator to generate the stress wave, and the other one installed on the center beam was utilized as a sensor to detect the stress wave signal. The function of each PZT transducer on the test specimen is shown in Table 2. The type of the PZTs used in the test specimen is PZT-5H and each PZT transducer has a diameter of 7.4 mm and a thickness of 1.3 mm. We used epoxy to bond the PZT patches on the specimen.

During the fatigue loading experiment, to simulate the effect of the fatigue damage on the stiffness of the test specimen, the dial indicators were installed to monitor the vertical displacement of the center beam, as shown in Figure 4. After finishing a certain number cycle loading, the displacements of the center beam were measured under static load. In the test process, the load distribution beam was installed on the location between the mid-spans of two side spans in the specimen. As a result, the dial indicator at the CB/SB2 joint and CB/SB3 joint cannot be installed. Hence, the displacements of the CB/SB2 joint and CB/SB3 joint could not be obtained, and the average displacement at the joint of the test specimen was only the average displacement of DI2 and DI6.

### 3.2. Experimental Setup and Procedures

**Figure 5:**
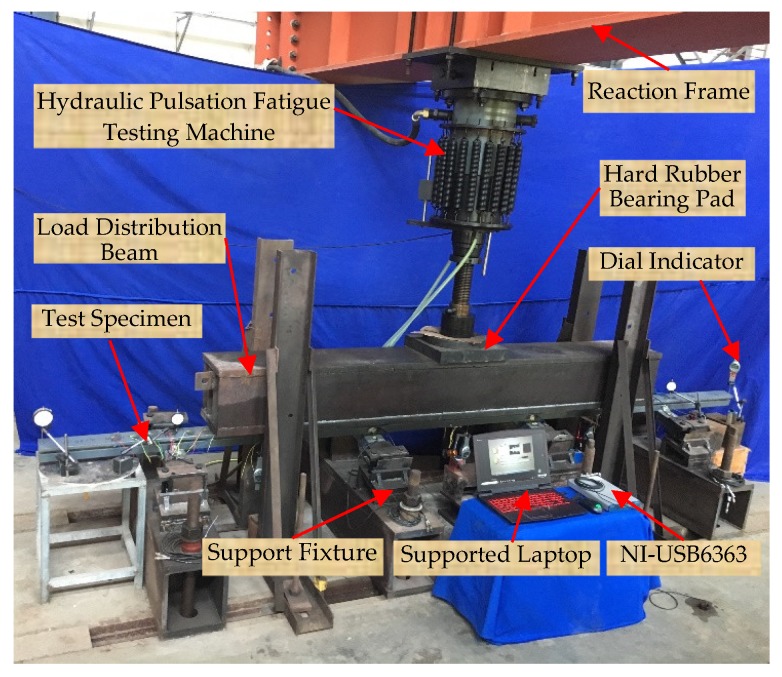
Photo of the experimental setup.

The experimental setup includes one full-scale CB/SB assembly specimen with PZT transducers, one reaction frame, one hydraulic pulsation fatigue testing machine, one hard rubber bearing pad, one load distribution beam, seven dial indicators to measure the vertical displacement, one data acquisition board NI-USB 6363, and one supported laptop, as shown in Figure 5. The hydraulic pulsation fatigue testing machine was mounted on the reaction frame, and the test specimen was attached on the support fixtures. The loads generated by hydraulic pulsation fatigue testing machine were transferred to the test specimen through the load distribution beam. The reaction frame and the support fixtures were mounted on a strong floor. Since the support fixtures were also capable of providing the supporting and securing of the specimen during the test, the support fixtures were fabricated as precisely as possible to avoid additional stress generated in the specimen as a result of the support fixtures misalignment.

**Figure 6:**
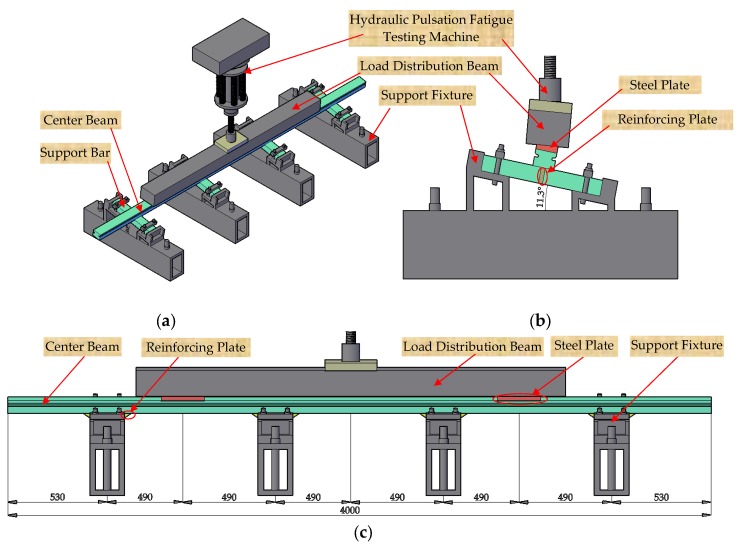
Loading configuration of the test specimen (unit: mm). (**a**) Three-dimensional view. (**b**) Left view. (**c**) Front view.

According to the guidelines of the *NCHRP 402 Report* [54], both vertical and horizontal load ranges shall be applied to the test specimen simultaneously in the ratio of 5:1. Thus, the support fixtures of the experimental setup were designed to make the specimen inclining at 11.3° from the horizontal plane to obtain the vertical and horizontal load ranges in the above given ratio, as shown in Figure 6. Loads were generated by the hydraulic pulsation fatigue testing machine and were transferred by the load distribution beam to the test specimen. The distance between the two load points of the center beam, which were located in the center of each outer span, was 1960 mm, as shown in Figure 6c. One steel plate and one hard rubber bearing pad were placed between the hydraulic pulsation fatigue testing machine and the load distribution beam for ensuring the good contact performance. Moreover, two steel plates installed between the load distribution beam and the center beam can perform a good contact condition.

A designed swept sine wave signal (steady-state signal) was used as the excitation signal for the PZT actuator transducers bonded on the support bar. The frequency range of the swept since wave signal is from 100 Hz to 150 kHz. The amplitude and the duration of the swept sine wave are 10 V and 1 s, respectively. In the tests, the predetermined input signal (excitation signal) was directly programed and sent to the PZT actuator transducer from the NI-USB 6363. Since the data acquisition board NI-USB 6363 has the function of transmitting and receiving the signals simultaneously, the output signals from the PZT sensor transducers mounted on the center beam can be recorded using the NI-USB 6363 in the test. And in the static testing, in order to monitor the effect of fatigue damage on the stiffness of the test specimen, the dial indicators mounted on the test specimen were used to measure the displacement of the center beam.

Two series of tests were performed on the specimen. The first series were static tests. The second series involved fatigue tests. Load ranges were monitored continuously throughout the test. Before the formal loading test, to eliminate the initial defect of the specimen and to ensure that the components of the specimen were in good condition and the loading equipment and test instruments could work normally, the pre-loading test of 20 kN was carried out. The experimental program of the specimen is shown in Table 3.

Static tests were performed to obtain the effect of the fatigue damage on the stiffness of the test specimen. The static test was divided into 12 levels of loading, the initial load was zero, the maximum load was 120 kN, and the increment of each load was 10 kN. The displacements of the test specimen were measured by the dial indicators under the different load levels in the static loading. Fatigue tests were applied in the form of a sine function, which the average load was 66 kN, the load amplitude was 54 kN, the minimum load was 12 kN, the maximum load was 120 kN, and the test frequency was 2.5 Hz, as given in Table 3, until the test specimen failure or the target number of 4 million cycles was reached. According to the target number of 4 million cycles in the fatigue test, the specimen included six stages: the 0th cycle (the healthy state), 0.8 million cycles, 1.6 million cycles, 2.4 million cycles, 3.2 million cycles, and 4 million cycles. When the fatigue tests reached a certain stage, the fatigue load was completely removed. We firstly checked the fatigue damage of the specimen with a digital magnifier. Then the PZT transducer enabled active sensing was performed and the signals of PZT transducers were recorded by the data acquisition system. Finally, we performed the static tests to measure the displacement of the specimen by the dial indicators. After finishing the above test process and verified the reliability of the test data, the fatigue tests would continue until the next stage was reached or the entire test was over.

## 4. Experimental Results and Discussions

### 4.1. Fatigue Damage Characteristics of the Specimen

**Figure 7:**
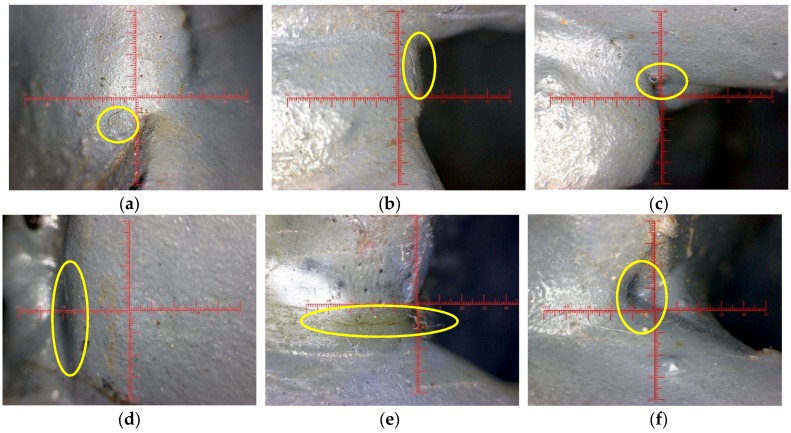

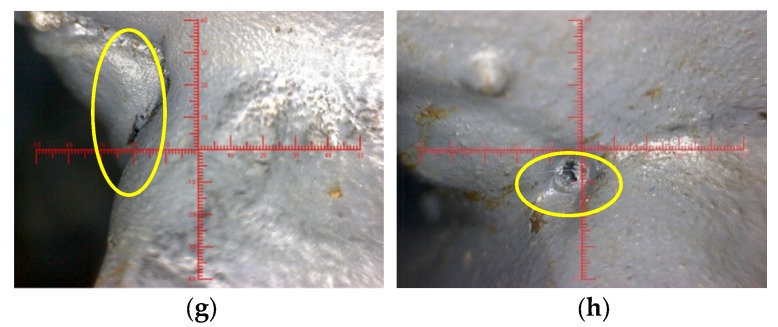
Partial enlargement photos of typical weld defect before the fatigue test. (**a**) South side of the CB/SB1 joint. (**b**) North side of the CB/SB1 joint. (**c**) South side of the CB/SB2 joint. (**d**) North side of the CB/SB2 joint. (**e**) South side of the CB/SB3 joint. (**f**) North side of the CB/SB3 joint. (**g**) South side of the CB/SB4 joint. (**h**) North side of the CB/SB4 joint.

The digital magnifier was utilized to detect the fatigue appearance damage in the test specimen. Before the fatigue test, all apparent weld defects were checked, which included undercut, overlap, pit, incompletely filled weld, and surface cracks. These defects weakened the welds and tended to generate stress concentration, which was the source of fatigue damage, and should be monitored. And the detection process was as follows: (1) connect the digital magnifier to the computer as a detection system; (2) aim the lens of the digital magnifier at the weld of the test specimen, and then rotate the focal length of the lens to make the appearance of the weld clear on the computer screen; and (3) if a weld defect is found, mark and take photos with a scale to calculate the size of the defects. There were eight welds to be monitored in the CB/SB joints of the test specimen, a typical defect in each weld was selected as the focus of the fatigue test, and the partial enlargement photos of typical weld defects are shown in Figure 7. In the entire fatigue experiment, except the defect of south side of the CB/SB3 joint developed into fatigue crack, no obvious expansion was found in other weld defects, and basically there is no change in the appearance.

**Figure 8:**
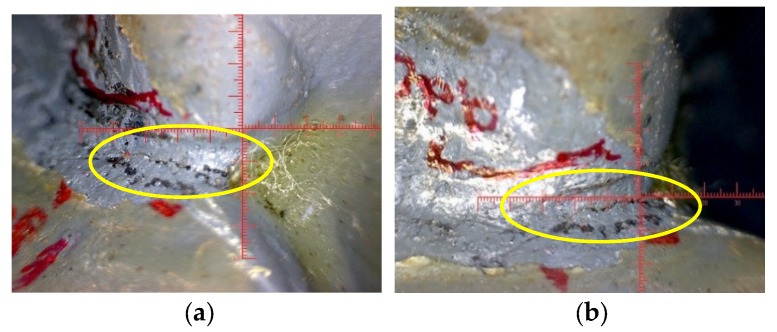
Diagram of fatigue cracks of south side of the CB/SB3 joint. (**a**) 3.2 million cycles. (**b**) 4.0 million cycles.

For typical weld defect of south side of the CB/SB3 joint, the photo before the fatigue experiment is shown in Figure 7e, the fatigue crack diagram when fatigue load reaches 3.2 million cycles is shown in Figure 8a, the fatigue crack diagram when fatigue load reaches 4.0 million cycles is shown in Figure 8b. Comparing the three pictures, it can see that when fatigue load reaches 3.2 million cycles, the typical weld defect develops into obvious fatigue crack. Under the action of fatigue load, it can be seen that the fatigue crack opens, closes, opens, again and again. The fatigue crack originates in the mid-depth of the weld throat in the south side of CB/SB3 weld joint, and the crack typically grows along the longitudinal axis of the center beam.

There is a ruler in each defect photo mentioned above. And the ruler needs to be calibrated for each defect photo due to different magnifications of the lens in the process of using. The results after calibration show that the length of the typical weld defect of south side of the CB/SB3 joint before the fatigue test is 4.55 mm. When fatigue load reaches 3.2 million cycles, the weld defect develops into the fatigue crack, and its length is 4.95 mm. When fatigue load reaches 4.0 million cycles, the fatigue crack grows along the longitudinal axis of the center beam, and its length reaches 6.72 mm.

### 4.2. Stiffness Characteristics of the Specimen

In the full-scale CB/SB assembly fatigue test, the *NCHRP 402 Report* observed that support bar stiffness has little effect on center beam moments [54]. Therefore, the CB/SB joints are considered as the fixed supports of the center beam, and it can be seen that the center beam of the test specimen has four fixed supports. Due to the load distribution beam blocking the installation of the dial indicators of CB/SB2 joint and CB/SB3 joint, the displacements of CB/SB2 joint and CB/SB3 joint cannot be obtained. We only use the average displacement of the measurement point DI2 of CB/SB1 joint and the measurement point DI6 of CB/SB4 joint as the average displacement of all the four fixed supports, as shown in Figure 4. The displacement of the center beam is defined as positive upward and negative downward. In the following analysis, the displacement of each measurement point should subtract the average displacement of the fixed supports.

**Figure 9:**
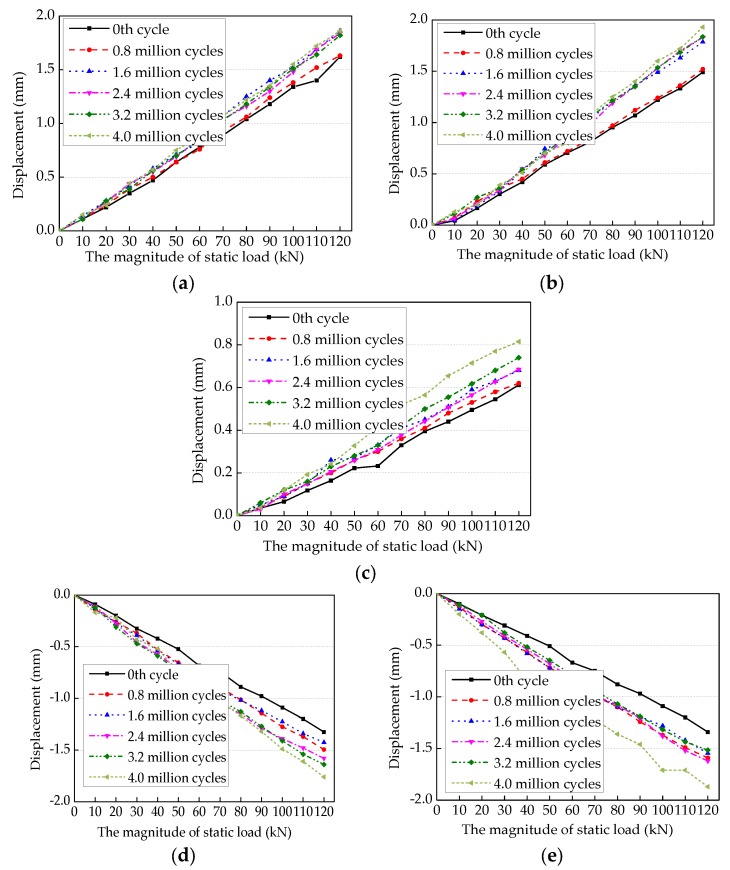
Effect of fatigue cycles on static load—displacement curves. (**a**) Measurement point DI1. (**b**) Measurement point DI7. (**c**) Measurement point DI4. (**d**) Measurement point DI3. (**e**) Measurement point DI5.

Under the static loading, there are three measurement points of displacement vertical upwards in the test, and they are the measurement points DI1 and DI7 of two ends of the center beam and the measure point DI4 of mid-span of the center beam. For the measurement points DI3 and DI5 of side-span of the center beam as the loading location, the displacement is downward. Effect of fatigue cycles on static load—displacement curve of measurement points is shown in Figure 9. It can be seen that when the fatigue cycles increase, fatigue damage of the weld gradually accumulates. This causes the support bar to be less restrictive to center beam and the overall stiffness of the test specimen decreases, resulting the increased displacement of center beam under the same static load. For the measurement points DI1 and DI7 of the center beam, when the fatigue cycles are between 0.8 million cycles and 1.6 million cycles, the displacements of the measurement points DI1 and DI7 increase obviously under the same load, as shown in Figure 9a,b. This indicates that the fatigue damage of CB/SB1 joint and CB/SB4 joint is very small before 0.8 million cycles, while the maximum of fatigue damage is between 0.8 million cycles and 1.6 million cycles. As a result, no visible fatigue cracks occur in the CB/SB1 joint and CB/SB4 joint during the fatigue loading process.

For the measurement point DI3, there is a significant change between 0th cycle and 0.8 million cycles and between 1.6 million cycles and 2.4 million cycles, as shown in Figure 9d. The displacement of measurement point DI3 is affected by fatigue damage of CB/SB1 and CB/SB2 joints, and the fatigue damage of CB/SB1 joint is mainly between 0.8 million cycles and 1.6 million cycles. Thus, the displacement change of DI3 is mainly due to the influence of CB/SB2 joint.

For the measurement point DI5, there is a significant stiffness change between the 0th cycle and the 0.8 million cycles and between the 3.2 million cycles and the 4.0 million cycles, as shown in Figure 9e. The displacement of measurement point DI5 is affected by fatigue damage of CB/SB3 and CB/SB4 joints, and the fatigue damage of CB/SB4 joint is mainly between the 0.8 million cycles and the 1.6 million cycles, thus the displacement change of DI5 is mainly due to the influence of CB/SB3 joint. This indicates that CB/SB3 joint has obvious fatigue damage between the 0th cycle and the 0.8 million cycles, and the fatigue damage develops into fatigue crack at the 3.2 million cycles and extended obviously until the end of the test, resulting in more increase in the displacement of measurement point DI5 between the 3.2 million cycles and the 4.0 million cycles. For the measurement point DI4, there is a significant stiffness change between the 3.2 million cycles and the 4.0 million cycles, and in other cycles the stiffness change is uniform, as shown in Figure 9c.

### 4.3. Time Domain Analysis

**Figure 10:**
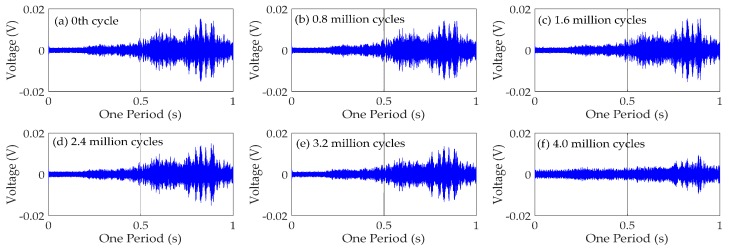
Time domain signals of PZT6 sensor for monitoring south side weld of CB/SB2 joint.

**Figure 11:**
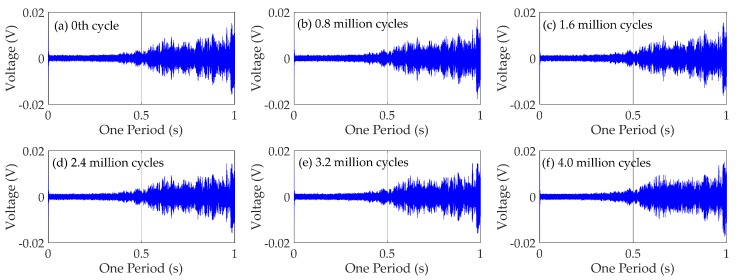
Time domain signals of PZT8 sensor for monitoring north side weld of CB/SB2 joint.

To obtain the time domain characteristics of the specimen, the following analysis takes CB/SB2 joint as an example. The time domain signals received by the PZT6 sensor for monitoring south side weld of CB/SB2 joint are shown in Figure 10. The time domain signals received by the PZT8 sensor for monitoring north side weld of CB/SB2 joint are shown in Figure 11. Each figure reflects the sensor response, at different fatigue cycles: (a) Before fatigue test, the 0th cycle; (b) 0.8 million cycles; (c) 1.6 million cycles; (d) reaches 2.4 million cycles; (e) 3.2 million cycles; and (f) 4.0 million cycles.

The results in Figure 10 and Figure 11 show that the amplitudes of the signal decrease with the increase of the fatigue load cycles in the test specimen. This is mainly due to the existence of weld defects, and these defects tend to generate stress concentration as the origin of fatigue damage. Therefore, the weld will inevitably suffer fatigue damage under the action of fatigue load, and the fatigue damage can continually accumulate with the increase of fatigue cycles. Hence, the signal received by the sensor will become weaker and weaker with the increase of fatigue load cycles.

**Figure 12:**
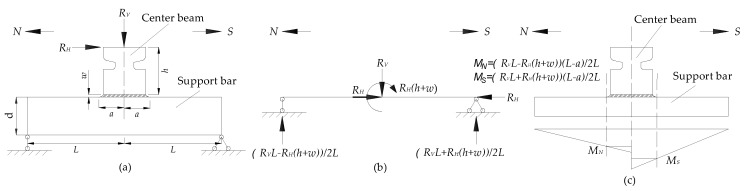
Mechanical analysis of CB/SB joint under the test load. (**a**) Details of the CB/SB joint. (**b**) Mechanical model of support bar. (**c**) Bending moment diagram of the support bar.

From Figure 10 and Figure 11, it also can be seen that the signal received by the sensor of south side of CB/SB2 joint changes more than that received by the sensor of north side of CB/SB2 joint. It can be explained by the following analysis of the CB/SB joint under the test load, as shown in Figure 12. The details of the CB/SB joint are shown in Figure 12a. The height of the center beam is *h*, the thickness of the weld is *w*, the distance of the weld toes is 2*a*, and the length and the height of the support bar are 2*L* and *d*, and the vertical force and the horizontal force of top side of center beam are *R_V_* and *R_H_*. According to the equilibrium equation, mechanical model of support bar is shown in Figure 12b, and bending moment diagram of the support bar is shown in Figure 12c. From Figure 12, we can see that the bending moment on the south side weld is larger than that of the north side weld. That is to say that the weld on the south side is more prone to fatigue damage than that on the north side. Therefore, the signal received by the sensor of south side of CB/SB joint changes more than that received by the sensor of north side of CB/SB joint due to the bending moment difference between south side weld and north side weld.

### 4.4. Wavelet Packet Energy Analysis

**Figure 13:**
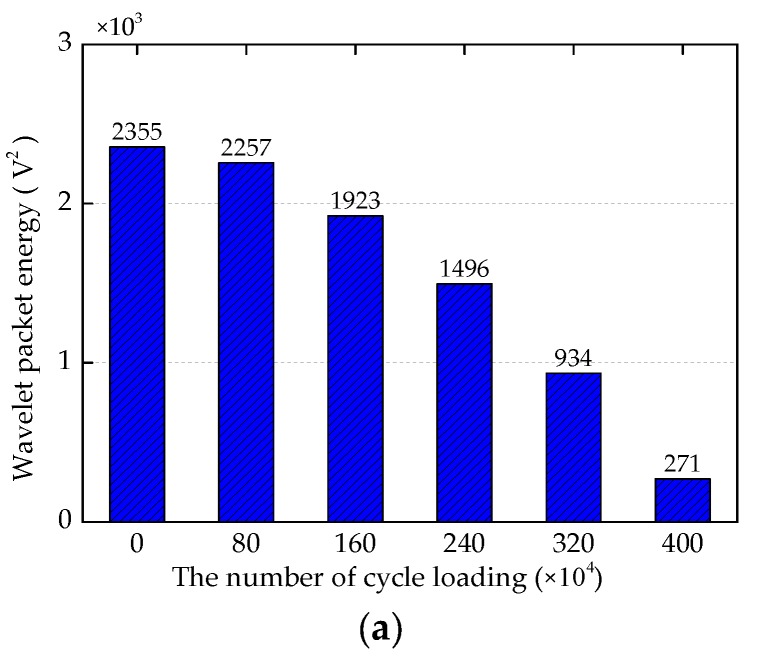

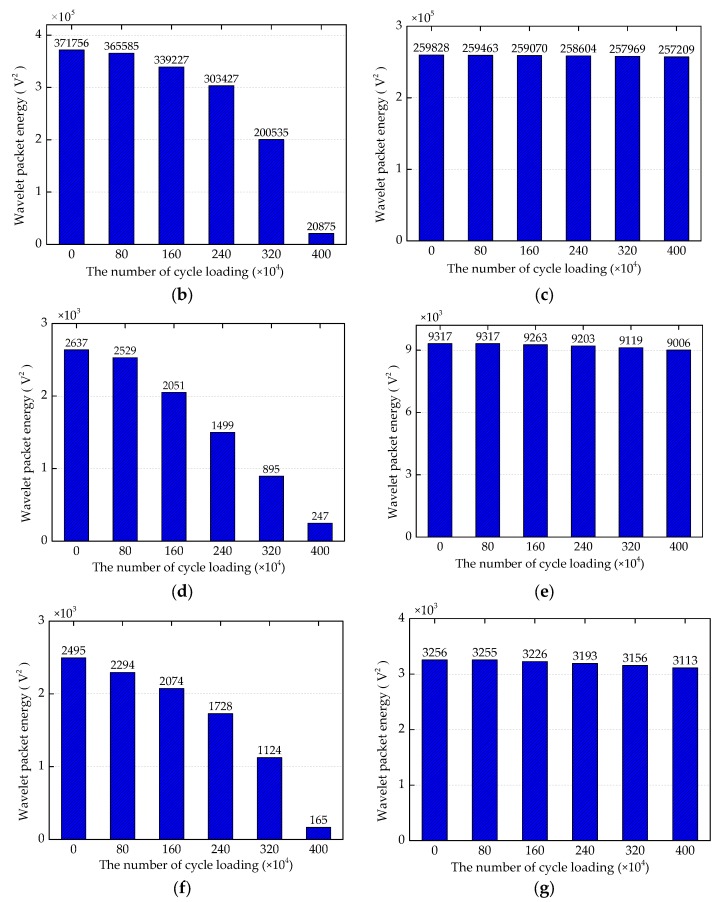
Wavelet packet energy analysis results of the specimen during the fatigue test. (**a**) The south side of CB/SB1 joint. (**b**) The south side of CB/SB2 joint. (**c**) The north side of CB/SB2 joint. (**d**) The south side of CB/SB3 joint. (**e**) The north side of CB/SB3 joint. (**f**) The south side of CB/SB4 joint. (**g**) The north side of CB/SB4 joint.

In the fatigue tests, the PZT4 sensor for monitoring the north side weld of CB/SB1 joint could not receive reliable signals, which may be caused by poor quality of lead wire soldered on the PZT4 patch. Therefore, the results from the PZT4 sensor will not be presented in the following analysis. In order to quantify the energy of the PZT signal during the fatigue test, the signal energy is computed by using the wavelet packet energy analysis. The wavelet packet energy analysis results of the specimen during the fatigue test are shown in Figure 13.

From these results, it can be seen the computed wavelet packet energy decreases with the increase of the number of cycle loading. This is mainly because the fatigue damage of the specimen increases gradually during the fatigue test, resulting in the decrease of the signal received by the PZT sensor. In these wavelet packet energy analysis figures, the received energy of the south side of the joint decreases much more than that of the north side for the test specimen. This is mainly because the test specimen is installed on the support fixtures of the experimental setup with an 11.3° inclination angle. With this inclination angle, the horizontal force *R_H_* produced on the top surface of the center beam is parallel to the support bar and points to the south of the support bar, and the vertical force *R_V_* produced on the top surface of the center beam is perpendicular to the support bar. Under the horizontal force *R_H_* and the vertical force *R_V_*, the bending moment of the south side of the CB/SB joint is greater than the bending moment of the north side. Therefore, the weld damage of the south side of the CB/SB joint is more than that of the north side of the CB/SB joint under the fatigue load. That is to say that the energy received by the PZT sensor of the south side decreases faster and more than that of the north side for the CB/SB joint of the test specimen under fatigue load. From the figures above, it can also be seen that wavelet packet energy of the north side for the CB/SB joint changes little, which shows that there is little damage to the weld of the north side for the CB/SB joint through the fatigue test. Therefore, the subsequent analysis refers to the weld of the south side of the CB/SB joint.

When the test specimen is in the state before the fatigue test, which represents the health state of the test specimen, the signals received by the sensors are used as the baseline signals. Therefore, compared with the results of the fatigue test, the maximum wavelet packet energy of the specimen occurs at the 0th cycle (the health state) of the test specimen. During the fatigue test, wavelet packet energy during the first 0.8 million cycles do not change significantly, which means that the damage of the specimen is extremely small. From the 0.8 million cycles to 3.2 million cycles, the slope of the wavelet packet energy decreases with the increase of number of loading cycles before reaching a saturation, which shows that the fatigue damage increases gradually with the number of cycle loading. Among them, wavelet packet energy of the south side of the CB/SB3 joint decreases the fastest, and this observation is consistent with the fact that only the weld on the south side of CB/SB3 joint eventually develops into a fatigue crack. After 3.2 million cycles, until the end of the fatigue test, the slope of the wavelet packet energy varying with the number of cycle loading grows rapidly, which indicates that the fatigue damage of the test specimen increases sharply, the fatigue crack length of the south side of the CB/SB3 joint increases from 4.95 mm at 3.2 million cycles to 6.72 mm at 4.0 million cycles. Therefore, the method using piezoceramic transducer enabled active sensing can detect the fatigue damage of the weld of the CB/SB joint in real-time.

In the future work, taking their advantages of stress wave generation and detection in a wide frequency range [60,61,62,63,64], we will use the PZT patch transducers as the time reversal mirror [65,66,67] to detect fatigue cracks in the CB/SB assembly specimen in connection with the time reversal method [68,69,70,71]. With both the spatial and temporal focusing property of the time reversal method, we believe that, the time reversal enabled active sensing method will provide more sensitive and accurate detection of fatigue crack.

## 5. Conclusions

This paper reports an experimental investigation to monitor fatigue damages in a full-scale CB/SB assembly specimen using surface-bonded piezoceramic transducer enabled active sensing. By the experimental verification, the fatigue damage of MBEJ was successfully monitored by using the stress wave based active sensing approach in real-time during the fatigue test. Experimental results illustrate that both the amplitudes and the wavelet packet energies of the signal received by PZT sensors decrease when the fatigue damage occurs. As the number of cycle loading increase, the damage of the CB/SB joint increase gradually, resulting the propagating stress waves are attenuated, so that the decrease of both the amplitudes and the wavelet packet energies of the received signal. Compared to the structure stiffness and wavelet packet energy, although the structural stiffness reduces as the number of fatigue loads increases, it could only focus on the whole specimen. Therefore, the structure stiffness cannot determine the fatigue damage degree of a certain weld. However, the proposed method can accurately detect the fatigue damage degree of any full-penetration weld in real-time, and has potentials to identify the initial fatigue damage occurrence for the structure of the MBEJ. A modular bridge expansion joints system is subjected to complex and variable random fatigue load in the actual application engineering, and the authors’ future work will be verified the reliability and the sensitivity of the proposed method by the additional full-scale CB/SB assembly specimen with different fatigue loads, such as variable amplitude fatigue load and random fatigue load. Furthermore, the time reversal method and electromechanical impedance (EMI) technique will be developed to increase the sensitivity of the proposed method.

## Figures and Tables

**Table 1:** Material properties of the test specimen.

Young’s Modulus (GPa)	Shear Modulus (GPa)	Yield Stress (MPa)	Ultimate Stress (MPa)	Density (kg·m^3^)	Poisson Ratio
200	76.9	350	450	7850	0.3

**Table 2:** The details of PZT transducers in the test specimen.

No.	Joint	Weld Side	Actuator	Sensor
1	CB/SB1	South	PZT1	PZT2
2	CB/SB1	North	PZT3	PZT4
3	CB/SB2	South	PZT5	PZT6
4	CB/SB2	North	PZT7	PZT8
5	CB/SB3	South	PZT9	PZT10
6	CB/SB3	North	PZT11	PZT12
7	CB/SB4	South	PZT13	PZT14
8	CB/SB4	North	PZT15	PZT16

**Table 3:** The experimental program.

Static Test			Fatigue Test				
Initial Load (kN)	Maximum Load (kN)	Increment of Each Load (kN)	Average Load (kN)	Load Amplitude (kN)	Minimum Load (kN)	Maximum Load (kN)	Frequency (Hz)
0.0	120	10	66	±54	12	120	2.5
